# First-Onset Psychosis Leading to Multiple Myeloma Diagnosis: A Case Report and Literature Review

**DOI:** 10.7759/cureus.43842

**Published:** 2023-08-21

**Authors:** Sean E Oldak, Anthony Maristany, William Ventura, Lujain Alhajji, Vanessa L Padilla

**Affiliations:** 1 Psychiatry and Behavioral Sciences, University of Miami Miller School of Medicine, Miami, USA; 2 Psychiatry and Behavioral Sciences, Jackson Memorial Hospital, Miami, USA; 3 Psychiatry and Behavioral Sciences, Universidad Tecnológica de Santiago, Santiago, DOM

**Keywords:** psychiatry, oncology, hematology, psychosis, multiple myeloma, cancer

## Abstract

Although multiple myeloma (MM) can cause various neurological complications, direct central nervous system (CNS) involvement is exceedingly uncommon and poorly understood. There has been one other reported case in the literature of a patient presenting with psychosis prior to diagnosis of MM. We present a case of a 58-year-old female with no history of psychiatric illness who presented to a behavioral health inpatient unit with paranoid delusions, multisensory hallucinations, and disorganized behavior in the days preceding her MM diagnosis. Due to hypercalcemia and altered mental status, she was transferred to an inpatient medical unit for further medical workup. Imaging revealed a sternal mass and diffuse lytic lesions. MM was confirmed. Her psychotic symptoms improved after one cycle of chemotherapy and steroids, treatment with aripiprazole, and resolution of hypercalcemia. Unlike other case reports where mental status changes have been described as consequences of already diagnosed MM, this patient's psychotic symptoms manifested prior to her MM diagnosis. While the exact pathophysiological mechanisms remain unclear, this case highlights a potential link between the sudden onset of psychosis and underlying undiagnosed MM. Healthcare providers need to be aware of this rare clinical presentation of psychosis in conjunction with MM.

## Introduction

Multiple myeloma (MM) is a rare and aggressive form of blood cancer derived from pathologic plasma cells in the bone marrow. Common symptoms include bone pain, anemia, fatigue, and frequent infections. MM can affect various organs and systems, posing significant challenges in diagnosis and treatment [1]. It has been previously documented that while the spectrum of neurological complications of MM is relatively diverse, direct central nervous system (CNS) complications due to MM are exceedingly rare and poorly understood [1]. One review found that CNS-infiltrated MM is a unique feature of a particularly aggressive and terminal disease state associated with high beta-2-microglobulin levels, high lactate dehydrogenase (LDH) levels, and secondary plasma cell leukemia [2,3]. Affected individuals often present with neurological symptoms such as diffuse cerebral dysfunction, cranial nerve palsies, spinal radiculopathies, pleocytosis, and elevated cerebrospinal fluid (CSF) protein content [2]. Individuals with MM commonly present with hypercalcemia, renal insufficiency, anemia, and bone lesions, with hypercalcemia often being a risk factor for altered mental status [4]. One case study reported altered mental status resulting from a relapsing kappa-light chain MM that led to hyperammonemic encephalopathy [4]. To date, the literature describes neuropsychiatric symptoms in individuals with late-stage MM, but there has been a paucity of reports of MM being diagnosed after the onset of neuropsychiatric signs, especially psychosis. We present an atypical case of a woman presenting with new onset psychosis as a preceding symptom of MM diagnosis. To our knowledge, this is only the second documented case of this phenomenon.

## Case presentation

A 58-year-old female with no past psychiatric history and a past medical history of well-controlled hypertension presented to a psychiatric hospital involuntarily brought in by police for paranoid delusions and disorganized behavior at home. The patient lived alone and was noted to be acting strangely by a neighbor who ultimately decided to call the police. As per law enforcement officers, the patient was found hallucinating inside her home, standing in a corner alone, seemingly speaking to persons who were not present. On initial presentation to the behavioral health hospital, she was alert, oriented to person, place, and date, distractible, with a tangential thought process, and superficially cooperative with the interview. She denied any active perceptual disturbances; however, she was seen overtly and grossly responding to internal stimuli, speaking to herself, and appearing to see and speak to people who were not actually in front of her. She was subsequently admitted to the adult inpatient psychiatric unit for psychiatric stabilization and management of new-onset psychosis.

**Figure 1:**
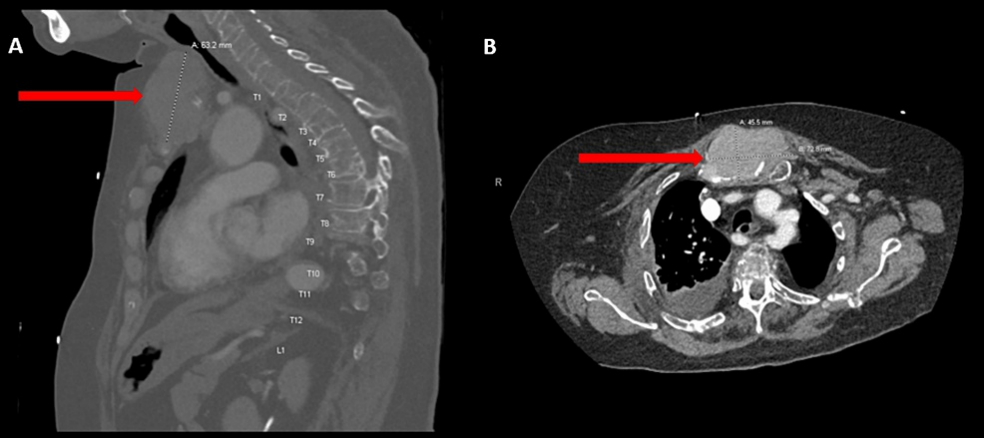
Large extensive soft tissue mass measuring 4.5 × 7.3 × 6.3 cm centered about the sternum on CTA; A: sagittal view, B: axial view CTA, computed tomography angiography

**Figure 2:**
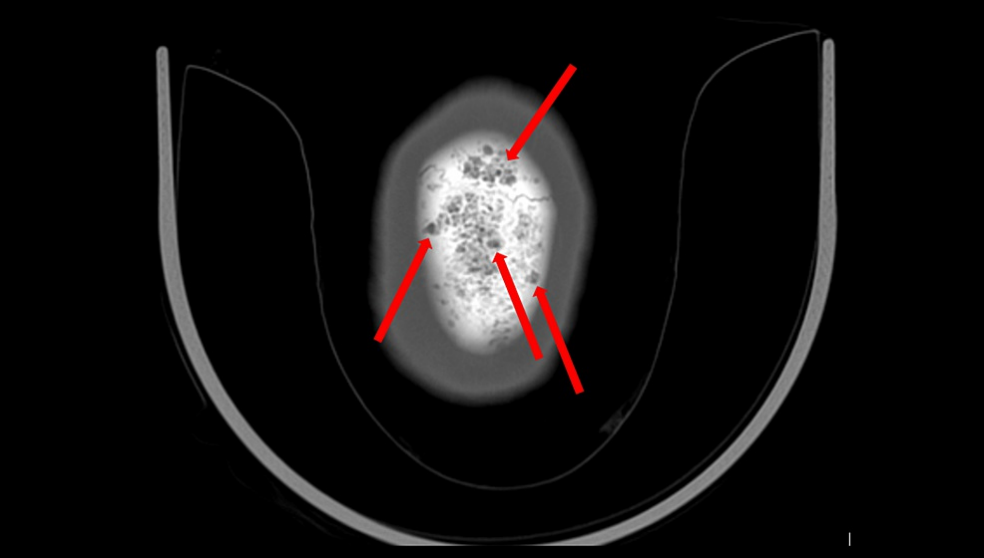
Extensive lytic appearance throughout the calvarium on CT CT, computed tomography

During the psychiatric admission where initial laboratory investigations were conducted (see Table 1), she was noted to be significantly anxious, guarded, labile, internally preoccupied, and impulsive, and exhibited mood dysregulation, disruptive behaviors (such as screaming and being uncooperative), thought blocking and disorganization, diminished emotional expression, and paranoia. She reported feeling suspicious and scared due to seeing a “man” in her peripheral vision, who was “grunting and mumbling” to her. She was treated with risperidone 2 mg at bedtime with minimal effect on her psychosis. On day four of her psychiatric admission, she developed progressive altered mental status, shortness of breath, and hyponatremia (Na=130 mmol/L; reference range=137-145 mmol/L). She was transferred to the medical emergency room (ER) for evaluation, where she was found to be tachycardic (up to 130 beats per minute) and fully alert and oriented. A complete physical examination was examined and was within normal limits. Laboratory results showed hypercalcemia (Ca=11.7 mg/dL and ionized Ca 1.56 mmol/L; reference range=8.4-10.2 mg/dL, 1.13-1.32 mmol/L, respectively). See Table 1 and Table 2 for further details regarding laboratory findings. Imaging revealed a sternal mass (see Figure 1) and extensive lytic-appearing lesions throughout the calvarium and skull base (see Figure 2). Besides this, the patient did not have any other CNS involvement, X-rays of shoulders and chest showed similar widespread lytic-appearing lesions, multiple right-sided rib fracture deformities, and wedge compression deformities of thoracic vertebrae. She was admitted to the intensive care unit for further workup and management of suspected MM. The hematology and oncology service was consulted, and a complete workup was done with the collection of serum protein electrophoresis (SPEP), urine protein electrophoresis (UPEP), free light chains, and immunoglobulins; all the results were consistent with MM diagnosis.

**Table 1:** Initial and subsequent routine laboratory values LDH, lactate dehydrogenase; BUN, blood urea nitrogen; AST, aspartate aminotransferase; ALT, alanine aminotransferase; CPK, creatine phosphokinase; WBC, white blood cells

Lab	Initial value	Subsequent value	Reference range
Glucose	98 mg/dL	85 mg/dL	74-106 mg/dL
Sodium	130 mmol/L	140 mmol/L	137-145 mmol/L
Potassium	5.5 mmol/L	3.8 mmol/L	3.6-5 mmol/L
Chloride	94 mmol/L	105 mmol/L	98-107 mmol/L
CO_2_	25 mmol/L	23 mmol/L	22-30 mmol/L
BUN level	12 mg/dL	11 mg/dL	7-17 mg/dL
Creatinine level	0.70 mg/dL	0.69 mg/dL	0.52-1.04 mg/dL
Calcium level	10.9 mg/dL	11.7 mg/dL	8.4-10.2 mg/dL
Total protein	7.3 g/dL	6.5 g/dL	6.3-8.2 g/dL
Albumin level	4.9 g/dL	4.2 g/dL	3.9-5 g/dL
Total bilirubin	1.4 mg/dL	1.4 mg/dL	0.2-1.3 mg/dL
AST	97 unit/L	38 unit/L	15-46 unit/L
ALT	51 unit/L	28 unit/L	9-52 unit/L
Alkaline phosphatase	110 unit/L	87 unit/L	38-126 unit/L
Magnesium level	1.3 mg/dL	1.6 mg/dL	1.7-2.2 mg/dL
CPK	238 unit/L	95 unit/L	35-230 unit/L
Lactate dehydrogenase	365 unit/L		120-246 unit/L
WBC count	7.4 x 10^3^/mcL	6.4 x 10^3^/mcL	4.0-10.5 x 10^3^/mcL
Hemoglobin	13.2 g/dL	12.6 g/dL	11.1-14.6 g/dL
Hematocrit	37.2%	37.7%	33.2-43.4%
Mean corpuscular volume	107.5 fL	112.5 fL	79.9-95.0 fL
Platelet	195 x 10^3^/mcL	201 x 10^3^/mcL	140-400 x 10^3^/mcL

**Table 2:** Immunology laboratory values for MM workup MM, multiple myeloma; IgG, immunoglobulin G; IgA, immunoglobulin A; IgM, immunoglobulin M

Lab	Value	Reference range
Albumin, urine	8.50 mg/dL	<5 mg/dL
Alpha 1 globulin, urine	1.30 mg/dL	0 mg/dL
Alpha 2 globulin, urine	5.70 mg/dL	0 mg/dL
Beta globulin, urine	4.60 mg/dL	0 mg/dL
Gamma globulin, urine	3.80 mg/dL	0 mg/dL
Serum total protein	5.9 g/dL	6.3-8.2 g/dL
Serum albumin fraction	3.70 g/dL	2.96-5.05 g/dL
Serum alpha 1 globulin fraction	0.50 g/dL	0.10-0.40 g/dL
Serum alpha 2 globulin fraction	0.60 g/dL	0.60-1.20 g/dL
Beta 1 globulin fraction	0.30 g/dL	0.34-0.52 g/dL
Beta 2 globulin fraction	0.30 g/dL	0.23-0.47 g/dL
Serum gamma globulin fraction	0.3 g/dL	0.6-2.0 g/dL
IgG	360.70 mg/dL	700.00-1600 mg/dL
IgA	35.80 mg/dL	70.00-400 mg/dL
IgM	12.90 mg/dL	40.00-230 mg/dL
Serum kappa light chains	101.5 mg/dL	170.0-370 mg/dL
Serum lambda light chains	41.7 mg/dL	90.0-210 mg/dL
Kappa/lambda ratio	2.434 ratio	1.350-2.650 ratio
Free kappa	9.10 mg/dL	0.33-1.94 mg/dL
Free lambda	0.68 mg/dL	0.57-2.63 mg/dL
Free kappa/lambda ratio	13.382 ratio	0.260-1.650 ratio
Beta-2-microglobulin, serum	5.63 mg/L	0.80-2.20 mg/L

The patient’s psychosis persisted, and the psychiatry team was consulted. On psychiatric evaluation, she was seen sitting in bed comfortably with no acute distress, alert and oriented to situation, place, day, and person; however, she appeared internally preoccupied and distractible. She reported continued visual hallucinations and remained paranoid. She was started on aripiprazole, which was titrated up to, and maintained at 20 mg oral tablet daily for psychosis, and 50 mg sertraline oral tablet daily for anxiety.

She was ultimately downgraded from the intensive care unit to the general medical floor and underwent bone marrow and sternal biopsy, which confirmed the diagnosis of stage III MM. Pathology showed an abnormal plasma cell population that represented ∼100% of the total plasma cells and 11% of the total white blood cells. Abnormal plasma cells showed cytoplasmic kappa light chain restriction with abnormal expression of CD19 (absent), CD45 (decreased), CD56 (uniform), and CD117 (increased) with normal expression of CD38 and CD138. Her hypercalcemia improved after treatment with IV normal saline, furosemide 20 mg twice daily, zoledronic acid 4 mg once, and calcitonin 300 units twice daily. She completed seven days of dexamethasone 20 mg daily and received one cycle of chemotherapy (cyclophosphamide and bortezomib). Her symptoms markedly improved and her psychosis resolved with this treatment. She was ultimately discharged home with the same psychotropic medication regimen she was stabilized on and with plans to follow up with hematology and oncology and psychiatry as an outpatient for further management. She was ultimately lost to follow-up.

## Discussion

While MM is rare, impaired cognition, particularly delirium, is common in patients diagnosed with it [5]. There has been growing insight into advanced-stage MM leading to secondary mania [6]. In another case of a 59-year-old woman with stage III MM, palliative chemotherapy treatment was effective in reducing manic symptoms and preventing disease advancement [6].

The combination of a one-cycle course of cyclophosphamide and bortezomib inpatient chemotherapy, in addition to a one-week steroid regimen, and use of an atypical antipsychotic, and improvement of her hypercalcemia with fluids, furosemide, zoledronic acid, and calcitonin was effective in improving our patient’s psychotic symptoms so that she could ultimately be discharged from the inpatient hospital setting. Our patient, however, while appearing internally preoccupied and easily distracted, was fully alert and oriented, with intact attention, and therefore did not meet the criteria for delirium and did not exhibit signs or symptoms consistent with mania. Furthermore, in other case reports and literature, neuropsychiatric symptoms seem to be described in patients with known diseases, while our patient’s psychotic symptoms preceded her diagnosis and discovery of her sternal mass and various lytic lesions throughout her torso and calvarium.

To our knowledge, this is only the second case report to describe psychosis as a preceding symptom to the diagnosis of MM. A previous case report published in 2014 described a 66-year-old woman patient who was also admitted to an inpatient psychiatric unit and had similar symptoms of psychosis that were recurrent and worsening [7]. This described patient did, however, have a history of previously treated major depressive disorder and right frontal lobe encephalomalacia secondary to a remote history of a gunshot wound [7]. Interestingly, this case report also had secondary psychosis that preceded the onset of MM by up to three months without signs of hypercalcemia, uremia, or hyperviscosity [7]. Our patient only presented with a past medical history of well-controlled hypertension and no past psychiatric history. Our case report differs also as hypercalcemia and hyponatremia were concurrent with her admission for the presentation of psychotic symptoms. Hypercalcemia is a common clinical manifestation in patients with MM as a result of prolonged renal insufficiency [8]. A prolonged period of severe (>14 mg/dL) subclinical hypercalcemia has shown to be associated with psychiatric symptoms, particularly psychosis [8,9]. Although a direct mechanism between hypercalcemia and psychosis has not yet been established, it is well known that calcium plays a role in monoamine metabolism in the CNS through modulation of dopaminergic and cholinergic neurotransmission which directly impacts mood and cognition [8].

While the pathophysiological elements still need to be explored, we believe that there may be a link between acute onset of psychotic symptoms, in a patient with no prior psychiatric history, and a MM diagnosis. Albeit rare, healthcare providers including hematology, oncology, and psychiatry providers should be aware of this potential clinical presentation of psychosis in tandem with an underlying undiagnosed MM. This is especially true as co-occurring psychiatric complications are associated with more healthcare utilization and increased cost as well as increased clinical complications of MM [10].

## Conclusions

This case report contributes to the growing understanding of neuropsychiatric manifestations in MM, shedding light on the importance of recognizing atypical presentations for prompt diagnosis and appropriate management. Further research is warranted to explore the complex interplay between MM and its potential neuropsychiatric complications, including the development of psychosis as a preceding symptom.
